# Erythrocyte Fatty Acid Profile, Mediterranean Diet and Asthma Severity in Childhood Allergic Asthma: Preliminary Findings from a Cohort Study in Spain

**DOI:** 10.3390/nu17071161

**Published:** 2025-03-27

**Authors:** Roser Ayats-Vidal, Isabela-Adelina Albiciuc, Carlota Bruch-Molist, Anna Cuartero-Gorjón, Begoña Cordobilla, Marina Pedrosa-Domínguez, Marta Susanna-Calero, Miguel García-González, Laura Valdesoiro-Navarrete, Helena Larramona-Carrera, Oscar Asensio-de la Cruz, Jesus Castro-Marrero, Joan Carles Domingo

**Affiliations:** 1 Pediatric Pneumology Unit, Pediatric Medicine Service, Hospital Sant Joan de Déu, 08950 Barcelona, Spain; 2 Pediatric Allergies, Immunology and Pneumology Unit, Pediatric Medicine Service, Institut d’Investigació i Innovació Parc Taulí (I3PT-CERCA), Parc Taulí Hospital Universitari, Universitat Autònoma de Barcelona, 08208 Sabadell, Spain; albiciuc.isabella@gmail.com (I.-A.A.); carlota.bruch17@gmail.com (C.B.-M.); a.cuartero.gorjon@gmail.com (A.C.-G.); marinapedrosadominguez@gmail.com (M.P.-D.); marta.susanna@gmail.com (M.S.-C.); mgarciago@tauli.cat (M.G.-G.); lvaldesoiro@tauli.cat (L.V.-N.); hlarramona@tauli.cat (H.L.-C.); oasensio@tauli.cat (O.A.-d.l.C.); 3 Department of Biochemistry and Molecular Biomedicine, Faculty of Biology, University of Barcelona, 08028 Barcelona, Spain; bgcordobilla07@ub.edu (B.C.); jesus.castro@vhir.org (J.C.-M.); 4 Division of Rheumatology, Research Unit in ME/CFS and Long COVID, Vall d’Hebron Hospital Research Institute, Universitat Autònoma de Barcelona, 08042 Barcelona, Spain; jesus.castro@vhir.org (J.C.-M.)

**Keywords:** mediterranean diet, erythrocyte fatty acids, omega-3 index, PUFAs, inflammation, asthma

## Abstract

**Background**: Allergic asthma incidence is increasing, probably due to the influence of the Western diet. Adherence to the Mediterranean diet (MedDi) and omega-3 fatty acids composition (*n*-3 PUFAs) may be linked to a lower prevalence and lower severity of childhood asthma; however, the association is inconclusive. This study aims to examine the relationship between adherence to the MedDi, asthma severity, and erythrocyte fatty acid profile in Spanish children with atopic asthma. **Methods**: This study was based on an ongoing single-center, prospective, cross-sectional cohort study involving 95 consecutively enrolled children from an outpatient tertiary referral center. Of these, 55 had atopic asthma and 40 were age-matched healthy controls. Blood samples were collected to analyze the erythrocyte fatty acid content. Participants’ demographic and clinical characteristics were recorded using validated self-reported outcome measures. Asthma severity and pulmonary function were assessed. **Results**: Asthmatics presented poorer adherence to the MedDi (*p* = 0.034) and lower *n*-3 PUFA levels (*p* = 0.019). Asthmatics with poor adherence to the MedDi were more likely to be overweight (*p* = 0.001) and to have moderate-severe asthma (*p* = 0.049); and lower *n*-3 PUFAs levels (*p* = 0.033). Children with mild asthma had higher *n*-3 PUFAs levels than those with moderate or severe asthma (*p* = 0.036). **Conclusions**: These findings highlight that adherence to the MedDi and a high erythrocyte fatty acid profile seem to have a protective effect in childhood asthma. Future well-controlled interventions should focus on the effects of MedDi patterns and *n*-3 PUFA intake on the primary prevention of childhood asthma.

## 1. Introduction

Asthma is the most common chronic disease in children and the most frequent cause of hospitalization and emergency room visits [1]. It affects approximately up to 334 million people worldwide, and its incidence has risen over the last three decades [2]. One of the explanations for this increase relates to changes in diet [3,4], with the expansion of the Western diet, characterized by a deficit of antioxidants and micronutrients including omega-3 polyunsaturated fatty acids (*n*-3 PUFAs) and vitamin D, and high levels of saturated fats with ultra-processed foods, processed meats, and pastries [5,6]. In contrast, the Mediterranean diet (MedDi), which includes a high consumption of vegetables, fruit, fish, legumes, nuts, and whole grains, is low in saturated fatty acids (SFAs) and rich in antioxidants and micronutrients [6]. It also has a high content of monounsaturated fatty acids (MUFAs), derived especially from olive oil, and *n*-3 PUFAs, derived mainly from oily fish [7,8,9].

The MedDi is a type of diet that is based on the dietary patterns of the countries surrounding the Mediterranean Sea, such as Spain, Italy, and Greece. This diet is characterized by being balanced and healthy, promoting the consumption of fresh foods and minimizing the consumption of processed foods [6]. The MedDi is one of the most widely studied diets in relation to the protection against chronic diseases, such as asthma [10,11,12] and allergic diseases in the pregnant mother and child [12,13,14,15,16]. Several meta-analyses have evaluated the effects of the MedDi on childhood asthma, but the results are not conclusive, probably due to the heterogeneous outcomes of the studies available; however, a protective trend has been described with respect to early wheezing and respiratory symptoms in the first years of the disease [17,18,19,20]. Lower dietary intake of *n*-3 PUFAs contributes to the increased morbidity related to asthma and allergic diseases in childhood [21].

The role of PUFAs in immunomodulation has been widely studied in relation to pulmonary conditions [22,23,24]. Linoleic acid (LA) is the most abundant *n*-6 PUFAs in the Western diet and is converted into arachidonic acid (AA), a precursor of both prostaglandin E2 and leukotriene B4, produced by mast cells and eosinophils [25]. Both prostaglandins and leukotrienes are potent bronchoconstrictors and exhibit proinflammatory properties in allergic diseases [24]. In contrast, alpha-linolenic acid (ALA), as a precursor of *n*-3 PUFAs, is metabolized into eicosapentaenoic acid (EPA), which inhibits AA and therefore reduces the production of pro-inflammatory eicosanoid mediators derived from *n*-6 PUFAs [26]. EPA and docosahexaenoic acid (DHA) are precursors of specialized pro-resolving lipid mediators (SPMs) such as maresin, protectin, and resolving families, which have important anti-inflammatory properties and have been considered therapeutic bioactive compounds for human health [26].

Recent studies have shown that an imbalance between pro- and anti-inflammatory molecules causes the exacerbation of chronic airway inflammation in asthma patients [27], and a higher intake of *n*-3 PUFAs has been linked to a significant protective effect against the incidence of asthma in children and adolescents [28,29,30,31]. However, to our knowledge, the association between PUFA intake and asthma has been studied in adults, but hardly at all in children [26].

This study aimed to compare the erythrocyte fatty acid content and the degree of adherence to the MedDi between a group of children with atopic asthma and a group of matched healthy controls. A secondary aim was to examine the relationship between these variables and the severity and control of asthma in the study population, as assessed by the lung function test.

## 2. Methods

### 2.1. Participants and Study Design

An ongoing single-center, prospective, cross-sectional cohort study was conducted in 55 children with mild to moderate or severe asthma (mean age: 10.96 ± 3.04 years old; 63% female) and 40 matched healthy controls (mean age: 10.92 ± 2.98 years old; 77% female) recruited consecutively between June and December 2022 from the outpatient tertiary referral center (Pediatric Pulmonary and Allergy Unit, Parc Taulí University Hospital, Barcelona, Spain).

Participants’ demographic and clinical characteristics were recorded using validated self-reported outcome measures, and the severity and control of asthma and pulmonary function were assessed.

Patients of either sex aged between 6 and 17 years were eligible for inclusion in the study. Cases were children diagnosed with asthma according to the doctor’s evaluation using the definitions: history of respiratory symptoms, such as wheeze, shortness of breath, chest tightness, and cough, that vary over time and in intensity, together with variable expiratory airflow limitation defined by GINA guidelines [32], sensitized to a respiratory allergen (prick test ≥ 3 mm or specific IgE levels ≥ 0.35 kU/L were considered positive), who did not have cystic fibrosis or other lung diseases, such as neuromuscular and thoracic cage disorders, that might affect the results. The control group were children being followed up at pediatric clinics who did not have asthma or any symptoms suggestive of asthma (recurrent wheezing, persistent cough, shortness of breath, and/or chest tightness), food allergy, allergic rhinitis, atopic dermatitis, cystic fibrosis, or other chronic inflammatory conditions.

### 2.2. Ethics Statements

The study protocol was approved by the Clinical Research Ethics Committee of the Parc Taulí Health Corporation (code 2021/5110, approved on 17 November 2021). Written informed consent was obtained from participants’ parents or legal guardians. Additionally, patients between 12 and 17 years old also provided signed informed assent.

### 2.3. Study Intervention and Data Collection

Most children were under the age of 12, and in these cases, parents were used as a proxy to fill out questionnaires. To assess the degree of adherence to the MedDi, the KIDMED questionnaire [33] was used, which comprises 16 questions about daily eating habits, with a simple “yes”/“no” answer. Questions with a negative connotation regarding MedDi are assigned a value of −1, and those with a positive connotation a value of +1. The sums of the test values are classified into three levels: (1) >8, optimal adherence to the MedDi; (2) 4–7, improvement is needed to adjust intake to Mediterranean dietary patterns; (3) <3, very low adherence to the MedDi.

In addition, the following general data were collected (age, sex, BMI, parents’ educational level, smoking partners, extracurricular sports, having been breastfed in infancy, and number of days a week oily fish is consumed). Overweight and obesity were determined according to the BMI percentile for age proposed by the WHO [34]. Overweight was considered when the volunteer presented a percentile between 85 and 95, while obesity was characterized as a percentile equal to or higher than 95. In order to perform statistical analysis, individuals with overweight and obese were grouped.

In the cases, the following clinical data were collected from the self-reported outcome measures:

1. Degree of asthma control was assessed using the CAN questionnaire (Asthma Control in Children), which asks a series of questions about asthma symptoms over the last 4 weeks, and the total score is obtained, ranging from 0 (absence of symptoms) to 36 points (maximum symptoms). A total average score equal to or greater than 8 on the CAN questionnaire is taken to indicate asthma control [35].
2. Degree of adherence to inhalers, using the TAI questionnaire (Inhaler Adhesion Test), in which adherence is considered good with a score of 50 or more, intermediate with a score between 46 and 49, and poor with a score of 45 or less [36].
3. Maintenance treatment indicated, in order to establish the severity of asthma according to the classification of the GINA guidelines [32], which assigns patients to one of four categories according to the therapeutic step: mild (step 1 or 2), moderate (step 3 or 4), and severe (step 5).
4. Comorbidities: allergic rhinitis, food allergy, and atopic dermatitis.
5. Lung function assessment with forced spirometry performed according to the American Thoracic Society guidelines [37]. The maneuver was performed in a sitting position, a disposable mouthpiece was attached to the spirometer, and a nose clip was used. The participants were instructed to inhale to total lung capacity and to exhale as hard and as fast as possible without a pause, and then a deep breath was inhaled to total lung capacity. The best of three technically acceptable tests were selected. We used the Sibelmed brand spirometer (Sibel, S.A.U, Barcelona, Spain), which provides standardized lung functions according to age, sex, and height in the reference population. The following parameters were analyzed: forced vital capacity (FVC), forced expiratory volume in the first second (FEV_1_), the ratio FEV_1_/FVC, and forced mid-expiratory flow (FEF_25–75%_). The results were expressed in liters, the percentage of the theoretical value expected for people of the same age, sex, and height in a reference population, and z-score, in accordance with the 2012 Global Lung Function Initiative (GLI) [38].

### 2.4. Blood Collection and Erythrocyte Fatty Acid Analysis

Blood samples were collected by venipuncture into tubes with anticoagulation (EDTA or citrate) from the participants. After removal of plasma by centrifugation, samples were stored at −80 °C until analysis. The composition of fatty acids in red blood cells was determined as methyl esters after a methylation reaction [39].

Erythrocyte membranes were extracted from 200 μL aliquots of erythrocyte suspensions, and fatty acids were converted to methyl esters by reaction with acetyl chloride at 100 °C for 60 min. A 1 M Tris-buffered saline solution was added to the erythrocyte aliquots, mixed by gentle inversion several times, and gently vortexed for 40 min to lyse the samples. The samples were then centrifuged, and the supernatant was removed. The erythrocyte membranes were washed three times with hypotonic saline. Finally, the pelleted cell membranes were resuspended in 100 μL of distilled water, and methylation was performed. Fatty acid methyl esters (FAMEs) were separated and analyzed by gas chromatography using a gas chromatograph mass spectrometer (GCMS-QP2010 Plus, Shimadzu, Kyoto, Japan), and the peaks of FAMEs were identified through electron ionization mass spectra using the NIST11 library and through GC retention times, comparing with a reference FAME mixture (GLC-744, Nu-Che Prep. Inc., Elysian, MN, USA). The results were expressed in relative amounts (molar percentage of total fatty acids) of duplicate sampling.

The panel of fatty acids analyzed included SFAs, MUFAs, *n*-3 PUFAs, and *n*-6 PUFAs. Among the *n*-3 PUFAs, the percentages of DHA were recorded. In addition, other fatty acid percentage ratios were calculated, including the omega-3 index (defined as (DHA + EPA)/total fatty acids) [40], *n*-6 PUFAs/*n*-3 PUFAs, and AA/DHA.

### 2.5. Statistical Analyses

The sample size was estimated according to type 1 (α) and type 2 errors (β) as 0.05 and 0.20 (power = 80%), respectively, based on the previous study [15]. Standard deviation (SD) and difference in mean or effect size (d) of RBC membrane fatty acids were considered as 1.04 and 0.78, respectively, as the key variables. Therefore, we needed 50 children with atopic asthma and 40 matched healthy controls as appropriate. Categorical variables are expressed as frequencies and percentages, and continuous variables as means and standard deviations (SD). All quantitative numerical variables were assessed for normality by the Shapiro–Wilk’s test. The chi-squared test was used for the comparison of categorical variables, and either the Student’s *t* test or the non-parametric Mann–Whitney U test was used for the comparison of quantitative variables, according to their conditions of application. Statistical significance was set at *p* < 0.05. SPSS version 25.0 was used for data analysis (IBM Corp., Armonk, NY, USA).

## 3. Results

### 3.1. Population Characteristics

Ninety-five children were included in the study, 55 cases and 40 controls. The mean age was 10.96 years, and 63.2% were girls. Table 1 shows anthropometric, demographic, and clinical characteristics of the study population. Children with asthma presented significantly lower adherence to the MedDi than healthy controls (KIDMED ≥ 8%: 40% vs. 63.6%, *p* = 0.034). Children with asthma presented a higher percentage of overweight than the controls, but the differences were not statistically significant; nor were there significant differences in the participation in extracurricular sports, parents’ educational level, or family smoking habits.

**Figure 1:**
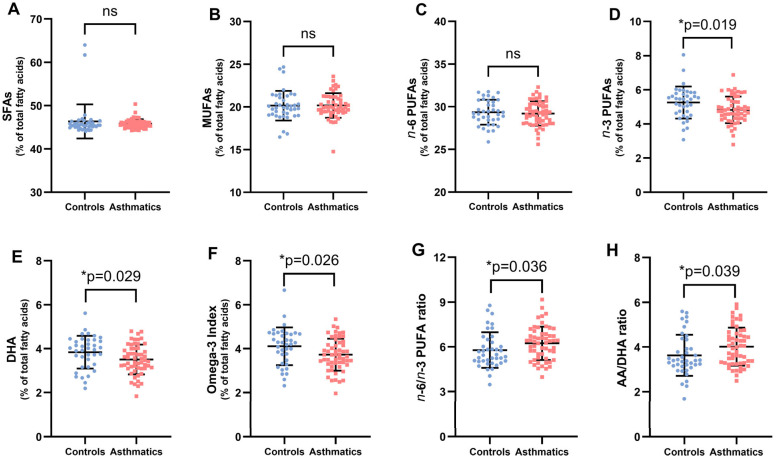
Baseline erythrocyte fatty acid composition and fatty acid ratios in the study population (panels (**A**–**H**)). Each dot represents a single participant in each group. Values are expressed as means ± SD of duplicate assays. Key: SFAs, saturated fatty acids; MUFAs, monounsaturated fatty acids; *n*-6 PUFAs, omega-6 fatty acids; *n*-3 PUFAs, omega-3 long-chain polyunsaturated fatty acids; DHA, docosahexaenoic acid; AA, arachidonic acid. *p*-values were calculated using the non-parametric Mann–Whitney U test to analyze the variations between asthmatics and controls. The level of significance was set at * *p* < 0.05; ns, not significant.

Table 1 and Figure 1 also display the fatty acid profile of children with atopic asthma and matched healthy controls. Children with asthma had significantly lower levels of *n*-3 PUFAs than healthy controls (4.82% vs. 5.25%, *p* = 0.019), and this is reflected in the significantly lower levels of DHA (3.50 ± 0.68) and the omega-3 index (3.73 ± 0.73), as well as significantly higher levels of the n6/n3 PUFA (6.24 ± 1.12) and AA/DHA ratios (4.02 ± 0.85).

### 3.2. Comparison Between Asthmatic Children with Good and Poor Adherence to the Mediterranean Diet

Table 2, which compares asthmatics with good and poor adherence to the MedDi according to the KIDMED questionnaire scores, shows that those with poor adherence had a higher BMI (*p* = 0.03) and a higher prevalence of moderate or severe asthma (69% vs. 39%, *p* = 0.041). Furthermore, the group with good adherence to the MedDi consumed more oily fish per week (oily fish ≥ 2 days/week: 35% vs. 3%, *p* = 0.005). The group of asthmatics with poor adherence to MedDi presented a higher percentage of children with poor adherence to inhalers, though the difference was not statistically significant (TAI ≤ 45: 22% vs. 0%, *p* = 0.082). Nor were any significant differences observed in comorbidities or lung function.

**Figure 2:**
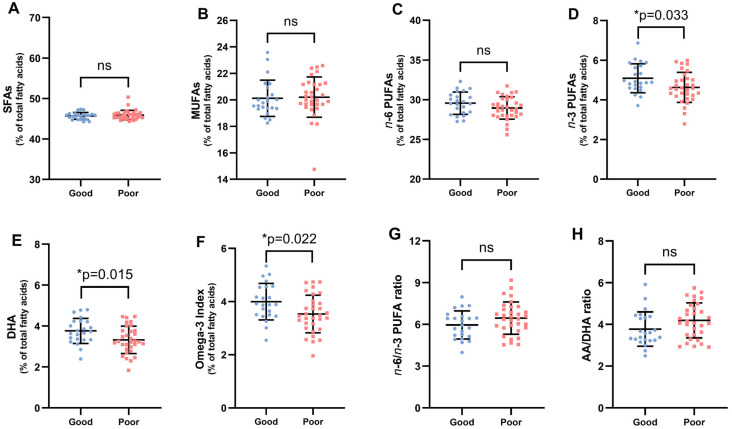
Erythrocyte fatty acid profile of children with asthma with good or poor adherence to the Mediterranean diet assessment by the KIDMED index (panel (**A**–**H**)). Each dot represents a single participant in each group. Values are expressed as means ± SD of duplicate assays. Key: SFAs, saturated fatty acids; MUFAs, monounsaturated fatty acids; *n*-6 PUFAs, omega-6 fatty acids; *n*-3 PUFAs, omega-3 long-chain polyunsaturated fatty acids; DHA, docosahexaenoic acid; AA, arachidonic acid. *p*-values were calculated using the non-parametric Mann–Whitney U test to analyze the variations of the adherence to the Mediterranean diet in children with asthma. The level of significance was set at * *p* < 0.05; ns, not significant.

With regard to the fatty acid profile, Table 2 and Figure 2 show that asthmatics with good adherence to the MedDi presented significantly higher levels of *n*-3 PUFAs, DHA, and a higher omega-3 index. No statistically significant changes were observed in SFAs, MUFAs, and *n*-6 PUFAs. Furthermore, asthmatics who reported consuming oily fish two or more days a week had higher levels of DHA (3.76 vs. 3.32, *p* = 0.02).

### 3.3. Comparison Between Children with Mild Asthma Versus Children with Moderate or Severe Asthma

Table 3 compares children with mild asthma with children with moderate or severe asthma. Notably, children with mild asthma had better adherence to the MedDi than those with moderate or severe asthma (KIDMED ≥ 8: 58% vs. 29%, *p* = 0.035). Children with mild asthma also had a lower proportion of children with a BMI percentile ≥ 85 (25% vs. 51.61%, *p* = 0.046), a higher percentage of parents with university degrees (*p* < 0.05), and a higher proportion of these children took part in extracurricular sports (92% vs. 55%, *p* = 0.007). Furthermore, as expected, children with moderate or severe asthma had poorer asthma control (CAN mean 1.54 vs. 4.9, *p* = 0.045).

**Figure 3:**
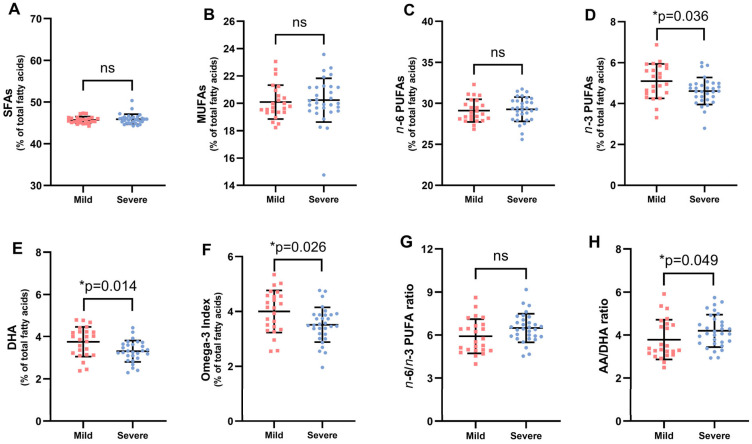
Erythrocyte fatty acid levels of children with asthma based on classification of asthma severity according to the Spanish Asthma Management Guidelines (GEMA) (panel (**A**–**H**)). Each dot represents a single participant in each group. Values are expressed as mean ± SD of duplicate assays. Key: SFA, saturated fatty acid; MUFA, monounsaturated fatty acid; *n*-6 PUFAs, omega-6 fatty acids; *n*-3 PUFAs, omega-3 long-chain polyunsaturated fatty acids; DHA, docosahexaenoic acid; AA, arachidonic acid. *p*-values were calculated using the non-parametric Mann–Whitney U test to analyze changes in asthma severity of the population with asthma. The level of significance was set at * *p* < 0.05; ns, not significant.

Both Table 3 and Figure 3 compare the fatty acid profile between these patients and show that children with mild asthma had a higher percentage of *n*-3 PUFAs (*p* = 0.036), DHA (*p* = 0.014), a higher omega-3 index (*p* = 0.025), and a lower AA/DHA ratio (*p* = 0.049).

## 4. Discussion

This study examines the effect of the MedDi and fatty acid profile in young people with atopic asthma. Children with atopic asthma presented poorer adherence to the MedDi than the control group, as also reported by Malaeb et al., who observed that occasional and daily MedDi consumption was significantly associated with a lower rate of current asthma in children [41]. Rice et al. also found that adherence to the MedDi among children was inversely associated with having asthma [42].

This study found that children with atopic asthma had lower levels of *n*-3 PUFAs, DHA, and omega-3 index than the control group. These results agree with those of Zhang et al., who found a significant association between the daily intake of *n*-3 PUFAs (assessed through a dietary questionnaire) and the reduction in the risk of asthma in children and adolescents, with a critical threshold observed at approximately 59 mg/kg/day [29]. In contrast, Almqvist et al. found that plasma *n*-3 PUFA and *n*-6 PUFA levels were not associated with wheezing, eczema, or atopy after 5 years of study in childhood asthma [43]. Talaei et al. did not find an association between the intake of EPA plus DHA from fish and the incidence of asthma in general, but among children with a common variant of fatty acid desaturase (FADS), those who had higher EPA and DHA intake in mid-childhood presented a lower risk of incident asthma in adolescence [44].

Our study highlights that those asthmatic children with good adherence to the MedDi have a lower proportion of moderate or severe asthma. Calatayud et al. carried out a comparative before-after study in children aged 1 to 5 years with criteria for childhood asthma who were enrolled in a 1-year program designed to promote the adoption of a MedDi and observed that during the study the use of inhaled corticosteroids fell markedly, from 3.92 ± 1.61 to 1.11 ± 1.09 times per patient per year, and that of inhaled bronchodilators fell from 4.14 ± 1.61 to 1.12 ± 1.40 [45]. It seems that the adoption of the MedDi may make a significant contribution to improving childhood asthma, reducing the degree of severity.

Furthermore, the present study demonstrated that asthmatic children exhibiting poor adherence to the MedDi (KIDMED < 8) presented a higher BMI. In addition, those with moderate or severe asthma had a higher proportion of overweight or obesity, which has been associated with a greater probability of suffering childhood asthma and with more severe and poorly controlled asthma [46].

There was also a close relationship between the MedDi and the fatty acid profile, since asthmatic children with good adherence to the MedDi had higher proportions of *n*-3 PUFAs, DHA, and omega-3 index. In addition, children who reported consuming oily fish two or more days a week had higher levels of DHA (3.76% vs. 3.31%). These results are consistent with Papamichael et al., who carried out a parallel randomized controlled trial comparing the consumption of a MedDi supplemented with two meals of 150 g of cooked fatty fish weekly (intervention) with the usual diet (control) in children (aged 5–12 years) with mild asthma and observed that, at 6 months, significant differences in DHA, PUFAs *n*-3, and *n*-6/*n*-3 PUFA ratio (*p* < 0.001) between the groups [30]. In addition, a meta-analysis suggests that the introduction of fish early in life (6–9 months) and regular consumption of all fish (at least once a week) reduces asthma and wheezing in children up to 4.5 years old, while fatty fish intake may be beneficial in older children [31].

This is why, independently of the adoption of a MedDi, the Scientific Committee of the Spanish Agency for Food Safety and Nutrition (AESAN) recommends consuming 3 or more servings/week of fish, prioritizing blue fish [47].

Regarding lung function, we did not observe significant differences in relation to the degree of adherence to the MedDi, unlike the study by Amazouz et al., who found that children with high adherence to the MedDi (according to both the KIDMED questionnaire and the Mediterranean Diet Score) had a higher FEV_1_ and FVC [48]; similarly, Romieu et al. also demonstrated that a higher adherence to a MedDi was significantly related to better lung function in asthmatic children [49]. A possible explanation as to why we did not observe any change in spirometry with MedDi adherence could be because most of the participants had normal lung function and well-controlled asthma.

In our study, we also demonstrated that, compared to their peers with moderate or severe asthma, children with mild asthma had higher percentages of *n*-3 PUFAs and DHA and a higher omega-3 index (*p* < 0.05) and a lower AA/DHA ratio (*p* < 0.05). Thus, asthmatic children with a better fatty acid profile tend to have milder asthma.

### Limitations and Strengths

The study has some limitations that should also be mentioned. The first is the small sample size and the fact that all the children were from a single outpatient tertiary referral center. Second, as in other epidemiological studies, the fact that participants’ adherence to the MedDi and their illness severity were assessed through self-report questionnaires may have introduced measurement errors due to recall bias and over-reporting.

Our findings are likely to be generalizable to the Spanish pediatric population with asthma; however, their applicability to healthy childhood populations may be limited.

Finally, whereas most studies have analyzed fatty acid composition in plasma/serum, our study focuses on erythrocyte membranes; serum or plasma fatty acid profiles reflect short-term dietary fat intake over some three weeks, but analysis of fatty acid composition from erythrocyte membranes is more useful because red blood cells last an average of 120 days in the blood. This longer lifespan provides a better representation of long-term dietary fatty acid intake and tissue conditions in childhood asthma [50].

## 5. Conclusions

In summary, this is the first study to find poorer adherence to a MedDi and *n*-3 PUFA consumption (especially DHA) in Spanish children with atopic asthma as compared to healthy children. They suggest a protective role against the development of symptoms and exacerbations in childhood asthma. These findings must be confirmed in large prospective randomized controlled trials that recommend MedDi adherence and high *n*-3 PUFA consumption for primary prevention (by maternal consumption during pregnancy) and focus on clinical symptom improvement in early childhood asthma. More research and clinical efforts should be focused on the future.

## Figures and Tables

**Table 1:** Demographic and clinical characteristics and erythrocyte fatty acid profile of the study population.

	Asthmatics (*n* = 55)	Controls (*n* = 40)	*p*-Value
Age (years)	10.96 (3.04)	10.92 (2.98)	0.23
Gender (male)	37 (67.27)	23 (57.50)	0.45
Body mass index (BMI, kg/m^2^)	18.64 (10.91)	18.9 (7.50)	0.45
BMI percentile ≥ 85	22 (40.00)	12 (30.00)	0.31
Extracurricular sports	39 (70.90)	23 (57.50)	0.26
KIDMED score ≥ 8	23 (41.80)	23 (57.50)	**0.034**
Eat oily fish ≥ 2 days/week	9 (16.36)	8 (20.00)	0.85
Breastfeeding	47 (85.45)	30 (75.00)	0.23
Smoking cohabitants	20 (36.36)	14 (35.00)	0.99
Mother with secondary or university education	47 (85.45)	36 (90.00)	0.18
Father with secondary or university education	39 (70.90)	30 (75.00)	0.89
SFAs	45.80 (1.06)	45.48 (0.836)	0.16
MUFAs	20.17 (1.45)	19.91 (1.44)	0.48
*n*-6 PUFAs	29.21 (1.43)	29.35 (1.46)	0.66
*n*-3 PUFAs	4.82 (0.77)	5.25 (0.94)	**0.019**
EPA	0.22 (0.08)	0.27 (0.17)	0.29
DHA	3.50 (0.68)	3.84 (0.75)	**0.029**
Omega-3 Index	3.73 (0.73)	4.12 (0.86)	**0.026**
*n*-6/*n*-3 PUFA ratio	6.24 (1.12)	5.78 (1.19)	**0.036**
AA/DHA ratio	4.02 (0.85)	3.63 (0.91)	**0.039**

Data are expressed as means (SD) for continuous variables and as numbers of cases (percentages) for categorical variables, as appropriate. *p*-values were calculated by Mann–Whitney U-tests for continuous variables and from chi-squared tests for categorical variables. Erythrocyte fatty acids are indicated as relative values (%), mean % of total fatty acids. Key: SFAs, saturated fatty acids; MUFAs, monounsaturated fatty acids; PUFAs, polyunsaturated fatty acids; AA, arachidonic acid; EPA, eicosapentaenoic acid; DHA, docosahexaenoic acid. Bold values denote statistical significance at a *p*-level < 0.05.

**Table 2:** Comparison between children with asthma with good and poor adherence to the Mediterranean diet based on the KIDMED score.

	KIDMED ≥ 8 (*n* = 23)	KIDMED < 8 (*n* = 32)	*p*-Value
Age (years)	9.96 (2.40)	10.31 (2.81)	0.58
Gender (male)	18 (78.26)	19 (59.37)	0.24
Body mass index (BMI, kg/m^2^)	18.49 (4.12)	21.83 (7.00)	**0.030**
BMI percentile ≥ 85	7 (30.43)	15 (46.87)	0.22
Atopic dermatitis	10 (43.47)	16 (50.00)	0.78
Allergic rhinitis	20 (86.95)	27 (84.37)	0.99
Food allergy	5 (21.73)	10 (31.25)	0.60
Moderate-to-severe asthma	9 (39.13)	22 (68.75)	**0.041**
CAN score	1.74 (2.34)	4.66 (6.23)	0.09
TAI score ≤ 45	0 (0.00)	7 (21.87)	0.08
Mother with secondary or university education	22 (95.65)	25 (78.12)	0.12
Father with secondary or university education	19 (82.60)	20 (62.50)	0.36
Eat oily fish ≥ 2 days/week	8 (34.78)	1 (3.12)	**0.005**
FVC			
%	98.14 (12.96)	98.75 (13.41)	0.18
Liters	2.57 (0.85)	2.58 (0.88)	0.39
z-score	0.045 (0.65)	0.85 (0.656)	0.17
FEV1			
%	93.66 (14.16)	98.15 (13.41)	0.43
Liters	4.24 (14.65)	4.05 (13.93)	0.47
z-score	0.99 (0.75)	0.95 (0.74)	0.75
FEF_25–75%_			
%	78.43 (23.35)	80.02 (23.65)	0.73
Liters	2.00 (0.71)	2.04 (0.70)	0.56
z-score	1.78 (4.04)	1.71 (3.85)	0.81
FEV_1_/FVC (%)	86.80 (9.86)	87.07 (9.69)	0.17
SFAs	45.67 (0.87)	45.89 (1.18)	0.61
MUFAs	20.12 (1.37)	20.21 (1.52)	0.36
*n*-6 PUFAs	29.56 (1.40)	28.95 (1.42)	0.15
*n*-3 PUFAs	5.09 (0.73)	4.63 (0.76)	**0.033**
EPA	0.23 (0.10)	0.21 (0.06)	0.66
DHA	3.76 (0.61)	3.32 (0.66)	**0.015**
Omega-3 Index	3.99 (0.69)	3.53 (0.71)	**0.022**
*n*-6/*n*-3 PUFA ratio	5.95 (1.01)	6.44 (1.16)	0.13
AA/DHA ratio	3.77 (0.82)	4.19 (0.84)	0.08

Data are expressed as means (SD) for continuous variables and as numbers of cases (percentages) for categorical variables, as appropriate. *p*-values were calculated by Mann–Whitney U-tests for continuous variables and from chi-squared tests for categorical variables. Erythrocyte fatty acid analyses are indicated as relative values (%), mean % of total fatty acids. Key: SFAs, saturated fatty acids; MUFAs, monounsaturated fatty acids; PUFAs, polyunsaturated fatty acids; AA, arachidonic acid; EPA, eicosapentaenoic acid; DHA, docosahexaenoic acid. Bold values denote statistical significance at a *p*-level < 0.05.

**Table 3:** Comparison between children with mild asthma and moderate or severe asthma.

	Mild Asthma (*n* = 24)	Moderate or Severe Asthma (*n* = 31)	*p*-Value
Age (years)	10.72 (3.07)	10.72 (3.07)	0.37
Gender (male)	17 (70.83)	20 (65)	0.37
Body mass index (BMI, kg/m^2^)	20.31 (6.22)	20.28 (6.34)	0.09
BMI percentile ≥ 85	6 (25.00)	16 (51.61)	**0.046**
KIDMED score ≥ 8	14 (58.33)	9 (29)	**0.035**
Eat oily fish ≥ 2 days/week	4 (16.66)	5 (16)	0.95
Atopic dermatitis	9 (37.50)	47 (55)	0.26
Allergic rhinitis	22 (91.66)	25 (81)	0.47
Food allergy	5 (20.83)	10 (32)	0.48
Smoking cohabitants	7 (29.16)	13 (41.93)	0.49
CAN score	1.54 (2.24)	4.90 (6.25)	**0.045**
TAI score ≤ 45	1 (4)	6 (19)	0.87
Extracurricular sports	22 (92)	17 (55)	**0.007**
Mother with secondary or university education	24 (90)	23 (74)	**0.035**
Father with secondary or university education	21 (88)	18 (58)	**0.035**
FVC			
%	98.90 (13.48)	98.05 (13.15)	0.42
Liters	2.53 (0.84)	2.59 (0.87)	0.11
z-score	0.85 (13.48)	0.85 (13.15)	0.64
FEV1			
%	94.52 (14.52)	93.50 (13.98)	0.81
Liters	4.25 (14.22)	4.17 (14.35)	0.11
z-score	0.97(0.75)	0.93 (0.72)	0.82
FEF_25–75%_			
%	79.39 (23.32)	79.11 (23.73)	0.75
Liters	1.98 (0.66)	2.02 (0.70)	0.19
z-score	1.78 (4.09)	1.73 (3.96)	0.79
FEV_1_/FVC (%)	87.37 (9.86)	86.49 (9.69)	0.28
SFAs	45.70 (0.82)	45.88 (1.22)	0.75
MUFAs	20.09 (1.24)	20.23 (1.60)	0.39
*n*-6 PUFAs	29.12 (1.38)	29.28 (1.48)	0.50
*n*-3 PUFAs	5.10 (0.84)	4.61 (0.66)	**0.036**
EPA	0.24 (0.10)	0.20 (0.07)	0.15
DHA	3.76 (0.70)	3.31 (0.51)	**0.014**
Omega-3 Index	4.00 (0.77)	3.52 (0.63)	**0.026**
*n*-6/*n*-3 PUFA ratio	5.91 (1.20)	6.49 (1.00)	0.06
AA/DHA ratio	3.78 (0.92)	4.20 (0.76)	**0.049**

Data are expressed as means (SD) for continuous variables and as the number of cases (percentages) for categorical variables, as appropriate. *p*-values were calculated by Mann–Whitney U-tests for continuous variables and from chi-squared tests for categorical variables. For erythrocyte FA analysis, data were obtained by gas chromatography-mass spectrometry and are indicated as relative values (%), mean % of total fatty acid. Key: SFAs, saturated fatty acids; MUFAs, monounsaturated fatty acids; PUFAs, polyunsaturated fatty acids; AA, arachidonic acid; EPA, eicosapentaenoic acid; DHA, docosahexaenoic acid. Bold values denote statistical significance at a *p* level < 0.05.

## Data Availability

Data set available on request from the authors: The raw data supporting the conclusions of this article will be made available by the corresponding authors on reasonable request.
